# Gene networks and the evolution of olfactory organs, eyes, hair cells and motoneurons: a view encompassing lancelets, tunicates and vertebrates

**DOI:** 10.3389/fcell.2024.1340157

**Published:** 2024-03-12

**Authors:** Bernd Fritzsch, Joel C. Glover

**Affiliations:** ^1^ Department of Biological Sciences, University of Nebraska Medical Center, Omaha, NE, United States; ^2^ Sars International Centre for Marine Molecular Biology, University of Bergen, Bergen, Norway; ^3^ Laboratory of Neural Development and Optical Recording (NDEVOR), Department of Molecular Medicine, Institute of Basic Medical Sciences, University of Oslo, Oslo, Norway

**Keywords:** transcription factors, gene networks, motoneurons, hair cells, eyes, olfaction

## Abstract

Key developmental pathways and gene networks underlie the formation of sensory cell types and structures involved in chemosensation, vision and mechanosensation, and of the efferents these sensory inputs can activate. We describe similarities and differences in these pathways and gene networks in selected species of the three main chordate groups, lancelets, tunicates, and vertebrates, leading to divergent development of olfactory receptors, eyes, hair cells and motoneurons. The lack of appropriately posited expression of certain transcription factors in lancelets and tunicates prevents them from developing vertebrate-like olfactory receptors and eyes, although they generate alternative structures for chemosensation and vision. Lancelets and tunicates lack mechanosensory cells associated with the sensation of acoustic stimuli, but have gravisensitive organs and ciliated epidermal sensory cells that may (and in some cases clearly do) provide mechanosensation and thus the capacity to respond to movement relative to surrounding water. Although functionally analogous to the vertebrate vestibular apparatus and lateral line, homology is questionable due to differences in the expression of the key transcription factors *Neurog* and *Atoh1/7,* on which development of vertebrate hair cells depends. The vertebrate hair cell-bearing inner ear and lateral line thus likely represent major evolutionary advances specific to vertebrates. Motoneurons develop in vertebrates under the control of the ventral signaling molecule hedgehog/sonic hedgehog (*Hh,Shh*), against an opposing inhibitory effect mediated by dorsal signaling molecules. Many elements of *Shh*-signaling and downstream genes involved in specifying and differentiating motoneurons are also exhibited by lancelets and tunicates, but the repertoire of MNs in vertebrates is broader, indicating greater diversity in motoneuron differentiation programs.

## Introduction

**FIGURE 1:**
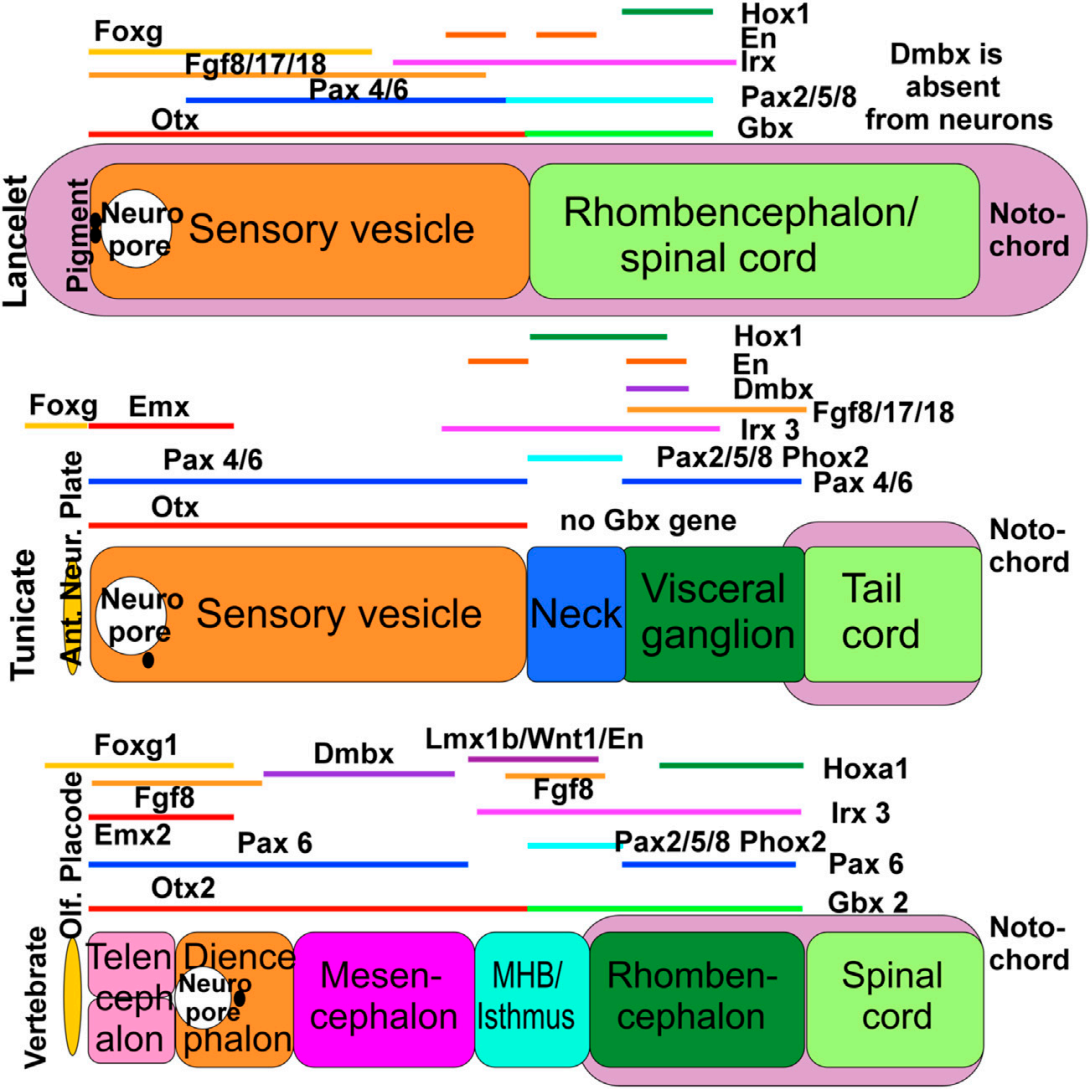
The evolution of gene expression in chordates, shown schematically here in lancelets, ascidian tunicates (represented by *Ciona*) and vertebrates. All three chordate groups exhibit partially overlapping expression of *Foxg, Emx, Otx, Irx*, and *Fgf* orthologs. In some cases the vertebrate gene names are used in all 3 panels for comparison, but we emphasize that these represent the corresponding gene orthologs in lancelets and tunicates (which may have different names in these taxa, particularly in cases where multiple orthologs exist in vertebrates). Lancelets do not express a *Dmbx* ortholog in the neural tube. In ascidian tunicates, *Dmbx* ortholog expression coincides with the caudal limit of *Irx3* ortholog expression and *Foxg* ortholog expression extends rostral to the neuropore. In vertebrates, *Dmbx* expression extends rostral to the *Irx3* expression domain to define the midbrain, while the forebrain and the olfactory placode are *Foxg1-positive.* In lancelets, *Gbx* ortholog expression overlaps with *Pax2/5/8* ortholog and most of *Irx3* ortholog expression. Ascidian tunicates have no *Gbx* ortholog but have a relationship between *Pax2/5/8 and Pax4/6* ortholog expression domains comparable to vertebrates. In vertebrates, the *Otx* expression domain abuts the *Gbx* expression domain. Together, these data show that certain gene expression domains are topographically conserved (*Hox, Otx*), whereas others show varying degrees of overlap (*Foxg, Dmbx*). It is conceivable that the evolution of nested expression domains of transcription factors is causally related to the evolution of specific neuronal features around the MHB. In vertebrates, the abutting domains of *Otx2* and *Gbx2* expression at the MHB stabilize the expression of Fgf8 (lower right), which in turn stabilizes the expression of *Wnt1* and engrailed (En1). Mutation of *Otx2, Gbx2, Fgf8, Lmx1b* or *Wnt1* eliminates the MHB. *Pax2/5/8* is also expressed at the MHB, whereas Dmbx expression starts immediately rostral to the MHB (in the midbrain) to later expand into the hindbrain and spinal cord. Experimental work has demonstrated that the development of vertebrate motor centers in the midbrain and upper hindbrain depends on the formation of the MHB. Ant. Neur. Plate; anterior neural plate boundary; MHB, midbrain/hindbrain boundary; Olf. Placode, olfactory placode. Modified after (Takahashi and Holland, 2004; Glover et al., 2018; Liu and Satou, 2019; Benito-Gutiérrez et al., 2021; Roure et al., 2023).

The comparative study of the nervous system and associated sensory structures can provide insight into the evolution of the molecular pathways governing their development and function. Chordates (lancelets, tunicates, and vertebrates) share several key structural features in this regard, including a notochord, a dorsal neural tube, and a dorsal opening of the rostral neural tube that forms the neuropore (Figure 1). Beyond this, there are additional similarities and some significant differences in the organization and development of sensory and efferent systems, which require elucidation at the molecular level. Recent genetic insights are beginning to facilitate evolutionary comparisons of these systems in the three chordate groups (Marlétaz et al., 2018; Poncelet and Shimeld, 2020; Marletaz et al., 2023; Roure et al., 2023).

Lancelets have only two main central nervous system (CNS) divisions, whereas tunicates have up to four (Takahashi and Holland, 2004) and vertebrates have at least five including the unique rostral extension comprising the bipartite telencephalon (Figure 1). The relative lengths of the notochord and neural tube differ; in lancelets the notochord extends beyond the rostral and caudal ends of the neural tube, in tunicates the neural tube extends rostrally beyond the notochord (Miyamoto and Crowther, 1985; Søviknes et al., 2007; Søviknes and Glover, 2008; Dong et al., 2009), and in all vertebrates the notochord ends near the midbrain-hindbrain boundary, so that the entire prosencephalon extends rostrally beyond the notochord (Welsch et al., 1998; Satoh et al., 2014; Witten and Hall, 2022; Fritzsch et al., 2023). In addition, distinct developmental lineages generate neural crest, placodes, eyes, and taste buds in vertebrates, but not in lancelets or tunicates (Moody and LaMantia, 2015; Holland, 2020; Elliott et al., 2022; Fritzsch and Martin, 2022; Adameyko, 2023; Zine and Fritzsch, 2023).

In terms of neuron numbers, some tunicates have a greatly miniaturized CNS, containing about 177 neurons in the larva of the ascidian *Ciona* (Ryan et al., 2018; Holland, 2020), and about 130 neurons in the appendicularian *Oikopleura* (Søviknes et al., 2005; Nishida et al., 2021). In contrast, the lancelet has about 20,000 neurons whereas vertebrates have a wide range of neuron numbers [from about 600,000 in lamprey up to 86 billion in humans; (Holland, 2020; Lent et al., 2012; Von Bartheld et al., 2016; von Twickel et al., 2019)].

Vertebrates generate a variety of cranial sensory structures derived from the ectodermal placodes, including (in rough rostro-caudal order) the olfactory, anterior pituitary, lens, trigeminal and otic and epibranchial placodes. In addition, the vertebrate neural crest gives rise to cells that delaminate from the dorsal neural tube to emigrate and establish a range of peripheral cell types and structures including sensory and autonomic ganglia. *Bona fide* placodes and neural crest are not found in lancelets and tunicates, although in tunicates two proto-placodal ectodermal domains have been identified that give rise to some peripheral sensory cells and may be homologous with the vertebrate placodes (Schlosser, 2015).

Tunicates as a group exhibit a wide range of genome sizes, up to 800–900 million base pairs (Mbp) in some species (Naville et al., 2019; Plessy et al., 2023), while other species have remarkably small and compact genomes (as low as 70–170 Mbp) (Denoeud et al., 2010). Lancelets have about 520 Mbp (Miyamoto and Crowther, 1985; Søviknes et al., 2007; Søviknes and Glover, 2008; Dong et al., 2009; Marlétaz et al., 2018) while vertebrates can have up to about 3,000 Mbp (Fodor et al., 2020; Holland, 2020; Marletaz et al., 2023). A major difference between lancelets and tunicates on the one hand and vertebrates on the other is the genome reshaping that followed two rounds of whole genome duplications (WGD) that occurred early in the vertebrate lineage, (Holland and Daza, 2018; Marlétaz et al., 2018; Simakov et al., 2022), with a third WGD having occurred in teleosts (Marletaz et al., 2023).

Many CNS patterning genes are common among chordates (Schlosser, 2015; Glover et al., 2018; Holland, 2020; Liu and Satou, 2020; Holland and Holland, 2021). However, their utilization differs across the chordate lineages. For example, *Otx* and *Gbx* interact to pattern the anteroposterior (AP) axis of the vertebrate CNS, but this interaction is only partly evident in lancelets, and the *Gbx* gene appears to be absent in *Ciona*, the most studied tunicate species (Figure 1). *Dmbx* is central in establishing the midbrain in vertebrates, but evidently plays a different role in lancelets and tunicates [Figure 1; (Takahashi and Holland, 2004; Glover et al., 2018)]. Expression of *Pax4/6* and *Pax2/5/8* and their orthologs in respectively more anterior and more posterior regions of the CNS differs in vertebrates, lancelets and tunicates (Figure 1). In vertebrates, WNT and BMP signaling contributes to anteroposterior patterning during neural induction [Figure 1; (Meinhardt, 2015; Kozmikova and Kozmik, 2020; Chowdhury et al., 2022; Roure et al., 2023)] and is also important for patterning dorsal structures of the brain stem and spinal cord, where it acts in concert with *Lmx1a/b* and *Gdf7* to induce the roof plate and the choroid plexus (Glover et al., 2018; Chizhikov et al., 2021; Elliott et al., 2022). Detailed analysis shows differences in the expression of *Wnt* genes of lancelets, tunicates and vertebrates (Somorjai et al., 2018; Roure et al., 2023), and the existence of a *Gdf7* ortholog is currently unclear in lancelets and tunicates (Kozmikova and Yu, 2017; Winkley et al., 2020; Fodor et al., 2021). The expression of *Fgf8/17/18* and their orthologs also differs markedly across the chordate lineages, relating to the prosencephalon and the midbrain/hindbrain isthmus in vertebrates (Watson et al., 2017; Jahan et al., 2021), to the cerebral vesicle in lancelets and to the visceral/caudal ganglion in tunicates (Figure 1). *Hox* genes have been essential for the evolutionary elaboration of the caudal neural tube that has created the rhombencephalon of vertebrates (Glover et al., 2018; Holland, 2020). Lancelets have a single *Hox* gene cluster, which has been duplicated twice to form 4 clusters in vertebrates (Holland and Holland, 2022), whereas tunicates have lost several *Hox* genes that are otherwise shared by lancelets and the Parahoxazoa [placozoans, cnidarians and bilaterians; (Seo et al., 2004; Ryan et al., 2010; Schultz et al., 2023)]. In vertebrates, *Atoh1* is expressed dorsally from the spinal cord to the brainstem, including the cerebellum (Bermingham et al., 2001). Cross-interaction between *Neurog1/2* and *Atoh1* (Fritzsch et al., 2006) influences development of the otic placode (*Neurog1, Atoh1*) and epibranchial neurons (*Neurog2, Atoh1*) in vertebrates (Fritzsch and Elliott, 2017; Zhang and Xu, 2023; Zine and Fritzsch, 2023). Neither an otic nor an epibranchial placode have been described in lancelets or tunicates, although a potentially homologous posterior proto-placode has been proposed in tunicates. Expression of the lancelet *Atoh1* ortholog has not been described in the CNS [only within mesoderm; (Chowdhury et al., 2022)], whereas expression of the tunicate *Atoh1* ortholog in tunicates is linked to the development of putative hair cells in the circumoral region (Tang et al., 2013) and their associated sensory neurons (Stolfi et al., 2015; Manni et al., 2018).

As in the CNS, the gene regulatory networks (GRN) governing the development of chemosensory, visual and mechanoreceptive cells and structures show some fundamental similarities but also important differences. Despite evidence for similar gene circuits in tunicate and vertebrate neural lineages (Ryan et al., 2018; Liu and Satou, 2020), absence of an early and broad expression of *Foxg* in tunicates contrasts with vertebrates, where *Foxg1* is essential for the development of olfactory organs, eyes, epibranchial neurons, and ears (Ermakova et al., 2019; Liu and Satou, 2019; Dvorakova et al., 2020; Zine and Fritzsch, 2023). Lancelets and tunicates have chemoreceptors, but these are not organized into an olfactory organ as seen in vertebrates. Lancelets, and tunicates that exhibit vision, have photoreceptors (Vopalensky et al., 2012) associated with supporting cells in primitive eye structures, but not a multilayered retina as in vertebrates. Indeed, developing tunicate eyes lack an *Atoh* pro-ortholog expression like the *Atoh7* expression that is critical for creating vertebrate retinal ganglion neurons (Ryan and Meinertzhagen, 2019; Wu et al., 2021; Fritzsch and Martin, 2022). Lancelets have putative mechanosensory cells but evidently none associated with acoustic mechanosensation (Lacalli, 2004; Wicht and Lacalli, 2005), and at least some tunicates develop cells with a morphology similar to vertebrate hair cells and with a distinct neuronal innervation (Rigon et al., 2013; Manni et al., 2018) (reviewed in Anselmi et al., 2024, in press). In contrast to these overt differences in sensory structures and cells, all three chordate taxa have motoneurons that innervate peripheral muscle used to generate body movements, although the manner in which this innervation is achieved can differ substantially (Søviknes et al., 2007).

In summary, substantial structural and cellular differences among lancelets, tunicates and vertebrates in the sensory systems for olfaction, vision and sensory/hair cell-mediated mechanoreception are beginning to be correlated with differences in the molecular networks governing the respective developmental specification and differentiation processes. On the efferent side, motoneurons are a highly conserved neuron type, yet exhibit a variety of target innervation modes. Lancelets, tunicates and vertebrates may share some elements of a common molecular framework for the generic specification of motoneurons, but this has been elaborated in vertebrates to create additional motoneuron subtypes.

We note that, to ensure distinction between gene and protein abbreviations, we use throughout this review the convention used for rodents (gene abbreviations are italicized with first letter in upper case and subsequent letters in lower case, protein abbreviations are not italicized, and all letters are in upper case).

## The olfactory system is prominent in vertebrates but less developed in lancelets and tunicates

The origin of G-protein coupled olfactory receptors (OR) genes can be traced back to the latest common ancestor of chordates, including lancelets, which have over 30 OR genes, comparable to lampreys (Niimura, 2009; Niimura, 2012). No orthologues of OR receptors have been found in tunicates, despite the presence of putative chemoreceptors in the oral region (Veeman et al., 2010; Kaji et al., 2016).

In vertebrates, the olfactory epithelium (OS) contains progenitors that give rise to distinct classes of olfactory sensory neurons (OSN), vomeronasal sensory neurons (VSN) and GnRH (gonadotropin releasing hormone)-expressing neurons that control the hypothalamic-pituitary-gonadal axis (Causeret et al., 2023). ORs are expressed by the OSN, whose axons projection to and establish glomeruli with second order neurons within the olfactory bulbs in the telencephalon (Fritzsch et al., 2019; Poncelet and Shimeld, 2020; Elliott et al., 2022; Zhu et al., 2022). Since their discovery (Buck and Axel, 1991), OR genes have been found in all vertebrates including fish, amphibians, reptiles, birds, and mammals. They comprise the largest vertebrate gene family, with up to 2,000 genes and hundreds of OR pseudogenes (Zhang and Firestein, 2002; Niimura and Nei, 2007; Niimura et al., 2020; Policarpo et al., 2023).

The olfactory epithelia (OE) and the olfactory bulb (OB) develop from the olfactory placode and the telencephalon, respectively (Fritzsch and Elliott, 2022; Imai, 2022). The olfactory placode is one of the cranial sensory placodes that give rise to several specialized sensory organs [OE, auditory and vestibular organs (Moody and LaMantia, 2015; Schlosser, 2021)]. A set of transcription factors, including *Eya/Six, Pitx, Otx2, Pax4/6,* and *Emx2* regulate the induction of the olfactory placode. Additionally, retinoic acid (RA), FGF8, SHH, and BMP4 secreted from adjacent mesenchymal cells define the axes of the OE and induce nasal cavity formation (Moody and LaMantia, 2015; Poncelet and Shimeld, 2020). These factors together trigger the upregulation of specific genes required for the generation of olfactory sensory neurons [e.g., *Sox2*, *Ascl1*, *Neurog1*, *Neurod1*, and *Foxg1*; (Panaliappan et al., 2018; Dvorakova et al., 2020; Bayramov et al., 2022; Roure et al., 2023)]. Specific microRNAs also play a critical role in the differentiation of these neurons (Kersigo et al., 2011).

**FIGURE 2:**
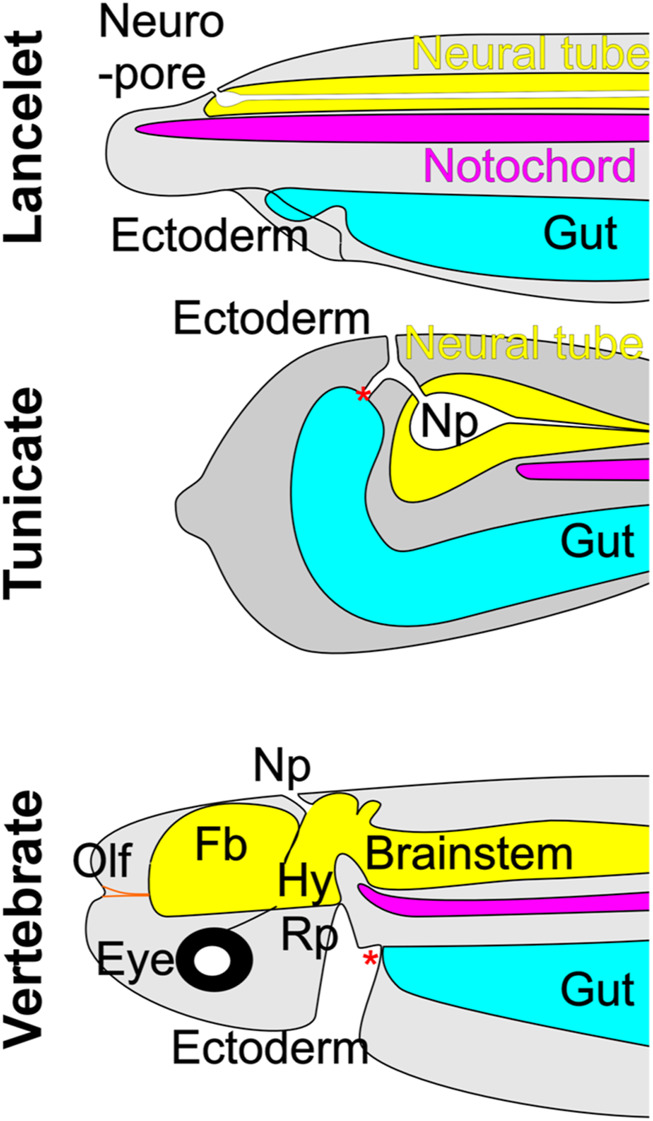
The neuropore (Np) in lancelets and tunicates (represented by the ascidian *Ciona*) forms an opening that connects the neurectoderm and the ectoderm. In vertebrates, the neuropore is transient and marks the point at which the neuroectoderm extends rostrally to form the forebrain (Fb), which appears to be unique to vertebrates. The olfactory placode (Olf), another vertebrate invention, develops rostral to the forebrain, and contains primary olfactory neurons that project to the forebrain. Neither lancelets nor tunicates have an olfactory system *per se* but have individual chemosensory cells that in lancelets likely express olfactory receptors. Note that in tunicates the neuropore has a collateral opening to the gut. In lancelets, the extension of the notochord prevents the formation of such a collateral opening. Vertebrates develop a structure in the embryonic oral ectoderm called Rathke’s pouch (Rp), which forms the hypophyseal placode (Hy) that exhibits a unique interaction with the CNS at the hypothalamus. * indicates the mouth opening. Modified after (Veeman et al., 2010; Winkley et al., 2020; Schlosser, 2021a).

Detailed analysis in *Ciona* has revealed the origin of the tunicate neuropore [Figure 2; (Veeman and Reeves, 2015)], which becomes confluent with the opening of the gut (Veeman et al., 2010) and is fused with the mouth orifice. This structural arrangement resembles that related to the embryonic origin of olfactory structures in vertebrates. Nevertheless, although tunicates possess oral chemoreceptors, they appear to lack *bona fide* homologs of the OE and OB (Niimura, 2012; Schlosser, 2021a). A similar anatomical relationship between neuropore and oral cavity is prevented in lancelets by the rostrally extended notochord [Figures 1, 2; (Ota and Kuratani, 2006)]. This structural intervention may be a contributing factor to the lack of a vertebrate-like olfactory system, since it could prevent inductive cell-cell interactions between gut and neural tube (Moody and LaMantia, 2015; Touhara et al., 2016; Schlosser, 2018; Holland, 2020; Poncelet and Shimeld, 2020). Although lancelets have no distinctive olfactory organ (Figure 2), they do have cells expressing OR genes near the mouth and along the lateral body wall (Niimura, 2012; Poncelet and Shimeld, 2020; Schlosser, 2021a).

GnRH is a marker across chordates for the anlage that give rise to the vertebrate adenohypophysis and olfactory structures and their potential homologs in lancelets and tunicates (Bassham and Postlethwait, 2005; Poncelet and Shimeld, 2020). The expression in tunicates of *Eya/Six*, which is necessary for olfactory development in mouse (Xu et al., 1997; Riddiford and Schlosser, 2016; Xu et al., 2021), initially suggested a potential evolutionary link to the vertebrate olfactory system (Bassham and Postlethwait, 2005). However, expression of *Foxg1* suggests that this likely only relates to peripheral olfactory structures. *Foxg1*, which is important for the development of the olfactory neural plate and olfactory bulb in vertebrates (Kersigo et al., 2011; Dvorakova et al., 2020), is only expressed at the anterior margin of the tunicate neural plate, where it is involved in the development of sensory neurons, but does not appear to be involved in the development of second-order neurons [Figure 1; (Panaliappan et al., 2018; Dvorakova et al., 2020; Liu and Satou, 2020; Benito-Gutiérrez et al., 2021; Zhang et al., 2021; Roure et al., 2023)]. Thus, despite some similarity between tunicates and vertebrates in the expression of GnRH, *Eya/Six* and *Foxg* (Roure et al., 2023), and of additional specific genes in GnRH-positive receptor cells (Touhara et al., 2016; Poncelet and Shimeld, 2020), it is unclear whether tunicates develop a homolog of the vertebrate olfactory placode or olfactory bulb (Cao et al., 2019). In this regard lancelets show greater similarity to vertebrates, as *Eya/Six* and *Foxg* are expressed in the lancelet sensory vesicle, part of the CNS (Figure 1).

In summary, neither lancelets nor tunicates possess a vertebrate-like olfactory system, but lancelets have OR-gene expressing receptors that may be homologous to vertebrate olfactory receptors, and tunicates develop GnRH-positive cells that may be homologous to vertebrate vomeronasal receptors. Only vertebrates have the extraordinarily extensive repertoire of OR genes and the elaborate synaptic interactions between olfactory sensory axons and central target neurons that form the glomeruli in the olfactory bulb. A more in-depth comparative review of olfactory system evolution can be found in (Poncelet and Shimeld, 2020).

## Evolving the visual system: from primitive photoreceptive structures in lancelets and tunicates to the eyes of vertebrates

Opsins are central to phototransduction and provide one basis for understanding evolution of the visual system in chordates (Lamb, 2020; Moraes et al., 2021; Roberts et al., 2022). Opsins evolved together with the regulatory gene *Pax6,* which drives the development of eyes across animal phyla, in many cases through interaction with retinoic acid (RA) signaling (Fuhrmann, 2010; Lamb, 2013; Schwab, 2018). A total of 21 opsin genes has been identified in lancelets (Pergner and Kozmik, 2017), and these code for highly variable opsin proteins, generating a diversity comparable to that in vertebrates. Comparison to the vertebrate opsin gene family has provided information on duplications and gene loss events in lancelets (Pantzartzi et al., 2018). By contrast, the tunicate *Ciona* has only 3 opsin genes, which have sequence similarity to the vertebrate visual opsins (Kusakabe et al., 2001; Terakita, 2005; Ryan and Meinertzhagen, 2019; Pisani et al., 2020).

**FIGURE 3:**
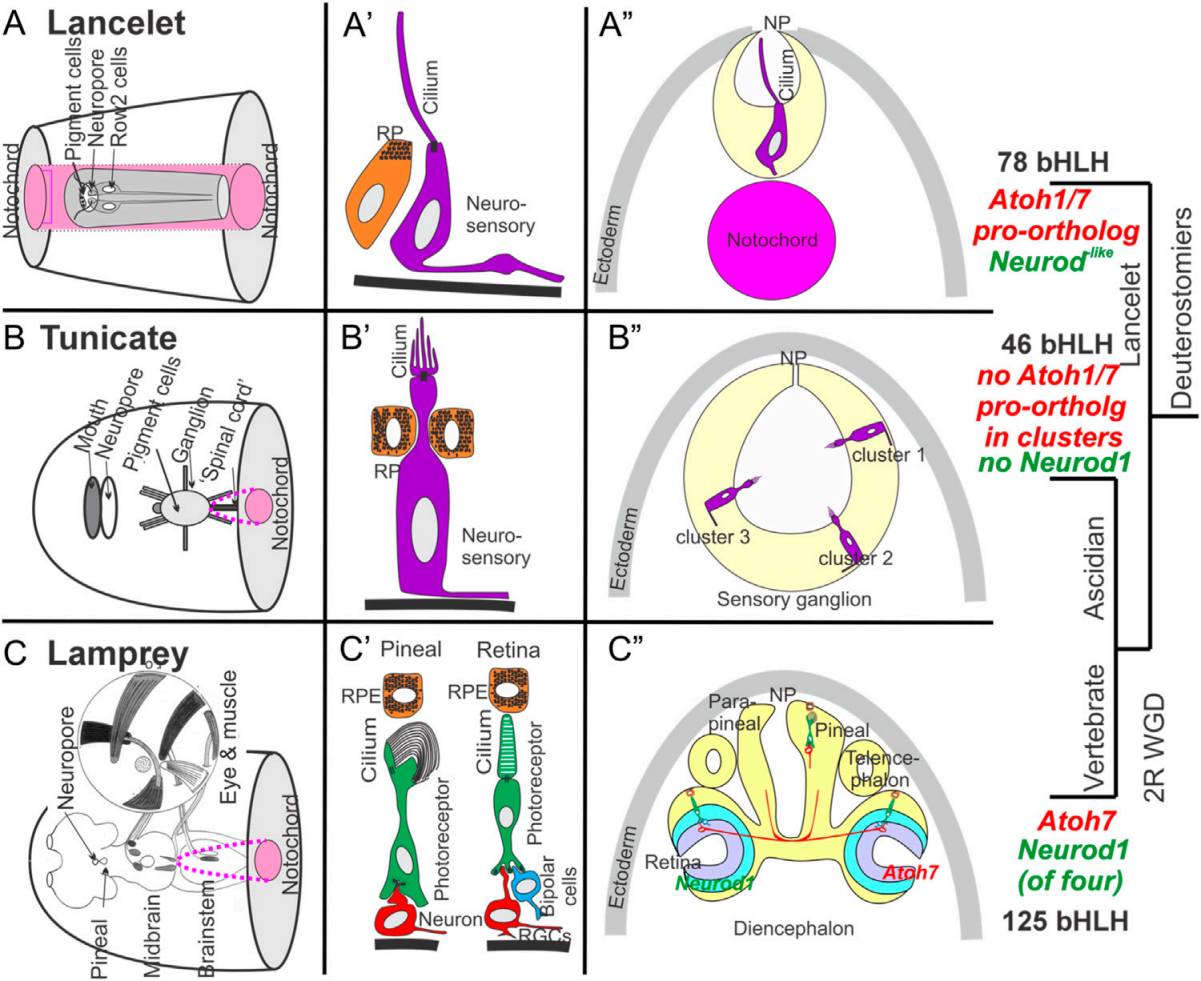
Comparison of lancelet, tunicate, and lamprey (vertebrate) photoreceptive organs. **(A)** Lancelets have a frontal eye **(A’)** with photoreceptor cells that contain visual pigments and that have a simple cilium but without lamellae. The cilium extends into the central lumen of the terminal sac towards the neuropore (NP) **(A”)**. Note that the notochord (pink) extends beyond the rostrum (transverse section view). **(B)** Ascidian tunicates have ciliated photoreceptors with an outer segment of lamellae **(B’, B”)**. Photoreceptors can be organized into as many as three distinct clusters, some associated with pigment cells in certain species **(B”)**. Note that the notochord (pink) does not extend to the level of the transverse section. **(C)** Lampreys have two photoreceptive organs: the pineal/parapineal and the retina **(C)**. Photoreceptor cells in both organs exhibit ribbon synapses **(C)**. Axonal projections to the brain arise either directly (pineal/parapineal) or indirectly via ganglion cells (RGCs) in the retina, which also contains bipolar, horizontal and amacrine cells that process visual inputs. The eye is moved by three sets of extraocular muscles (inset). Note that vertebrates express distinct bHLH genes (Atoh7, Neurod1, **(C’)**) in the developing eye. Expression of orthologs of these genes has not been demonstrated near the eyes in lancelets **(A’)** or tunicates **(B’)**. Atonal is required for eye development in flies and has evolved into multiple distinct Atoh, Neurod and Neurog genes in vertebrates. Modified after (Eakin and Kuda, 1970; Barnes, 1971; Eakin, 1973; Fritzsch and Collin, 1990; Fritzsch et al., 1990; Lacalli et al., 1994; Collin et al., 2009; Braun and Stach, 2017; Marlétaz et al., 2018; Suzuki and Grillner, 2018; Braun and Stach, 2019; Cao et al., 2019; Elliott et al., 2022; Elliott et al., 2021; Martin et al., 2021; Baker and Brown, 2018).

Potential evolutionary relationships between vertebrate eyes and pineal gland on the one hand and lancelet photoreceptive organs on the other have been reviewed in some detail recently (Pergner et al., 2020). Lancelets have four photosensitive organs, the frontal eye and the lamellar body (a presumed homolog of the vertebrate pineal gland), both of which contain ciliary photoreceptors, and the Joseph cells and the dorsal ocelli, both of which contain rhabdomeric photoreceptors. The frontal eye exhibits several similarities to the vertebrate retina, including specific gene expression domains comparable to vertebrate counterparts (Pergner and Kozmik, 2017). *Pax6*, considered a master regulator for eye formation, is expressed in the regions giving rise to all four of these photosensitive organs (Glardon et al., 1998). Cells associated with the frontal eye express the photoreceptor- and opsin-related transcription factors *Pax2/5/8*, *Six3/6*, *Otx*, *Mitf* as well as melanin synthesis genes [Figure 3; (Lamb et al., 2007)] and opsins (Vopalensky et al., 2012). Each photoreceptor cell in the frontal eye has a ciliary process that extends out of the neuropore, and an axonal projection to the CNS (Lacalli et al., 1994; Pergner and Kozmik, 2017) that may mediate further synaptic connections to motoneurons (Lacalli, 2018; Schlosser, 2021). In contrast to the frontal eye and lamellar body, the Joseph cells and dorsal ocelli express melanopsin, suggesting homology to vertebrate non-visual circadian photoreceptors (Gomez Mdel et al., 2009). Despite the clear presence of functional photoreceptors in all these structures, none of them approaches the complexity of constituent cell types found in the vertebrate retina and pineal gland. A potential explanation is the duplication of the *Atoh* pro-ortholog to create the *Atoh1 and Atoh7* genes in vertebrates, the latter of which is critical for creating vertebrate retinal ganglion neurons, and the lack of *Neurod* and *Otx* gene expression in the photoreceptive organs of lancelets, which is critical for creating vertebrate rods and cones (Ryan and Meinertzhagen, 2019; Wu et al., 2021; Chowdhury et al., 2022; Fritzsch and Martin, 2022).

In tunicates, ciliated photoreceptors associated with adjacent pigment cells have been described in many species, although appendicularian tunicates such as *Oikopleura* lack a visual organ entirely (Konno et al., 2010; Kourakis et al., 2019; Braun et al., 2020; Glover, 2020; Winkley et al., 2020; Olivo et al., 2021). In *Ciona*, the ocellus is made up of a cup-shaped pigment cell, 3 lens cells (not homologous to the vertebrate lens), and a number of photoreceptor cells that comprise 3 morphologically distinct groups (Olivo et al., 2021). Some of the photoreceptor cells are adjacent to pigment cells such that light detection by these is directional, and some are displaced from the lens cells and thus react primarily to unfocused light. The photoreceptor cells have an outer segment with multiple lamellae and each connects directly to neurons in the adjacent sensory vesicle that mediate photic responses (Lacalli and Holland, 1998; Konno et al., 2010; Razy-Krajka et al., 2012; Braun and Stach, 2019; Ryan and Meinertzhagen, 2019). In addition to opsins, *Ciona* also expresses the protein arrestin, a homolog of vertebrate β-arrestin which regulates opsin-based G-protein signaling to temporally curtail photoreceptor responses, suggesting that this function may be conserved from tunicates to vertebrates (Nakagawa et al., 2002; Kawano-Yamashita et al., 2011). In the salp *Thalia*, the blastozooid stage has a horseshoe-shaped set of pigmented cells adjacent to photoreceptor cells that are split among three cups that contain transparent transient lens cells and point in different directions (Barnes, 1971; Lacalli and Holland, 1998; Konno et al., 2010; Braun and Stach, 2017; Winkley et al., 2020). Small nerve branches connect these primitive eyes to the brain (Ryan et al., 2018; Braun and Stach, 2019).

*Pax6* is expressed in the ocellus of *Ciona* (Irvine et al., 2008), but as in lancelets, other genes associated with vertebrate retinal neuron specification are not, including *Atoh7*, *Neurod1 and Otx2* (Ryan and Meinertzhagen, 2019; Wu et al., 2021; Fritzsch and Martin, 2022). Another bHLH gene important for retinal development in vertebrates, *Atoh8* (Cao et al., 2019) is expressed in the sensory ganglion domain of *Ciona,* but is not clear whether it is eventually expressed in the ocellus [*Atoh8* is not expressed in the brain, only the retina, in vertebrates; (Negrón-Piñeiro et al., 2020a; Rawnsley et al., 2013; Divvela et al., 2022)]. It may be that the larger repertoire within certain key gene families generated through WGD in vertebrates (for example, duplication of an *Atoh* pro-ortholog to generate *Atoh1* and *Atoh7*) may have facilitated an increase in cell type and photoreceptive organ diversity (Fritzsch et al., 2010; Holland and Daza, 2018). A more limited gene repertoire might thus explain the less elaborate differentiation of eye-related cells and structures in tunicates.

*Pax6* is also expressed in the rostral CNS of appendicularian tunicates like *Oikopleura* (Cañestro and Postlethwait, 2007), but does not drive the formation of photoreceptors or any eye-like organ. Since retinoid signaling is an important factor promoting eye development in vertebrates (Cvekl and Wang, 2009), the evolutionary loss of all components of the retinoid signaling system in *Oikopleura* (Cañestro and Postlethwait, 2007) might explain the absence of photoreceptors in appendicularian tunicates despite expression of *Pax6*.

In summary, although lancelets, tunicates and vertebrates share opsins and arrestins as basic molecular components for phototransduction, and *Pax6* appears to be a common driver of photoreceptor development in all three (except for appendicularian tunicates), we lack enough information to relate the lancelet frontal eye and the multiple tunicate eyes to the bilateral eyes and pineal gland of vertebrates [Figure 3; (Lamb, 2013; Lamb, 2020; Fritzsch and Martin, 2022)]. The main difference is the absence of certain genes, particularly *Atoh7* and *Neurod1*, which are required for eye and pineal gland development in vertebrates. Moreover, the complete lack of photoreceptors despite *Pax6* expression in appendicularian tunicates requires a molecular explanation.

## Evolving hair cells and connecting them to the CNS

Hair cells are believed to have evolved as a retained phenotype from the common ancestor of choanoflagellates and metazoans. In this idea, the motility-enabling flagellum-microvilli complex has been converted to a mechanosensory device and the basic choanoflagellate cell type has given rise to a sensory cell within a synaptically-coupled neural circuit (Caicci et al., 2013; Fritzsch and Straka, 2014; Fritzsch and Elliott, 2017; Colgren and Burkhardt, 2022; Van Le et al., 2023). An important line of inquiry has been to determine whether lancelets and tunicates have hair cells that are homologous to the hair cells of vertebrates, which are found in the vestibular and cochlear sensory organs and in the lateral line neuromasts of vertebrates. For such homology to hold, it should be possible to find ciliated secondary sensory cells (sensory cells that do not have an axon, but receive synapses from primary sensory cells) in lancelets and tunicates.

**FIGURE 4:**
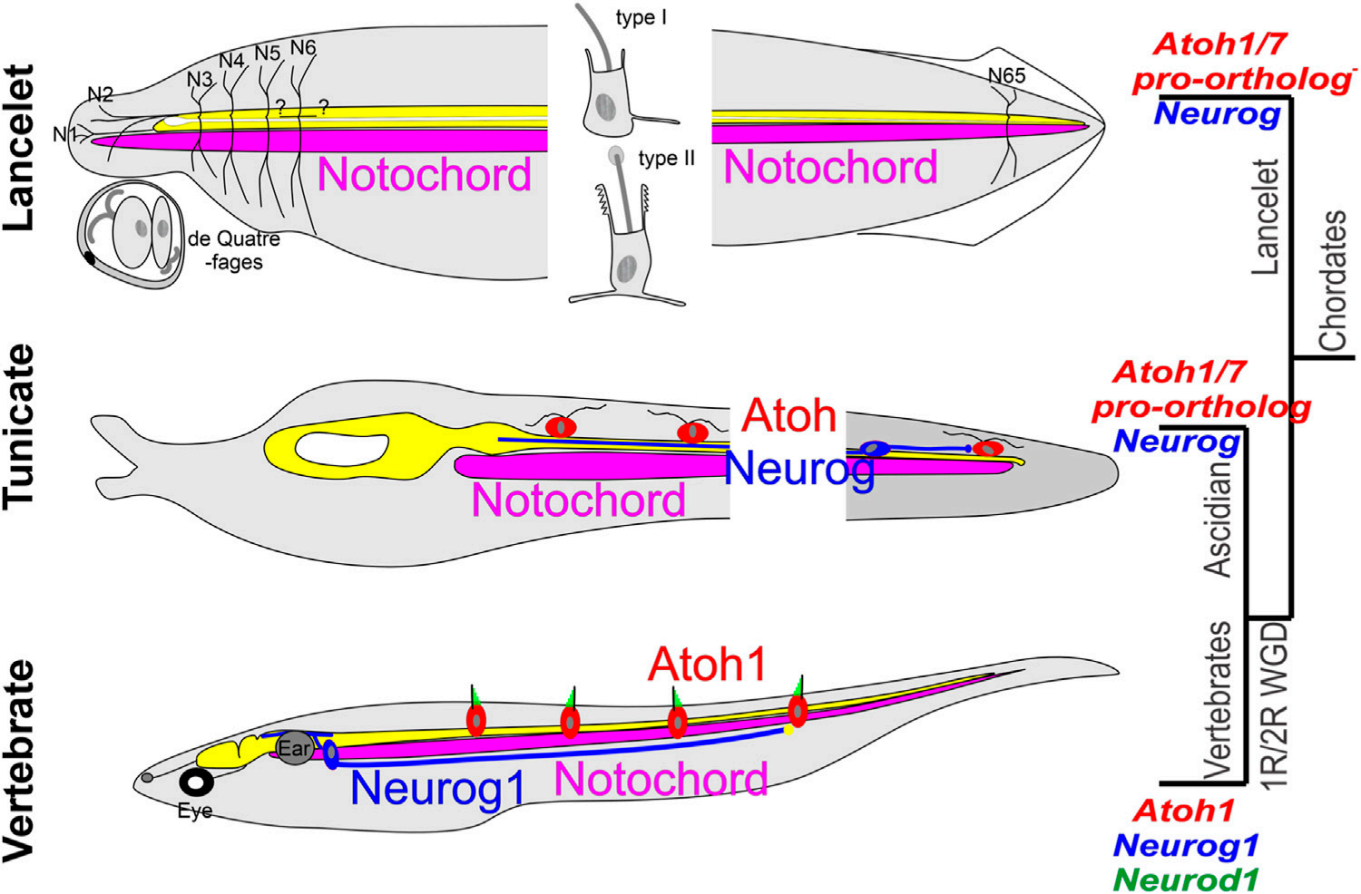
Comparison of some lancelet, tunicate, and vertebrate mechanosensory receptors. Lancelets have three types of ectodermally derived putative mechanosensory cells: the cells of the corpuscles de Quatrefages (associated with nerves 1 and 2), the type I epidermal sensory cell, which is a primary sensory cell with an axonal extension into the CNS, and the type II epidermal sensory cell, which is likely a secondary sensory cell that may be innervated by central neurons that have widespread axon terminations in the skin. Neither Neurog nor the Atoh1/7 pro-ortholog (NeuroD/Atonal-related gehe) have been linked to development of these cells (Neurog is only expressed in the notochord, expression of NeuroD/Atonal-related has not been described in the CNS). The ascidian tunicate Ciona has putative mechanosensory cells in the epidermis that have axons that can extend into the CNS (and thus appear to be primary sensory cells) or are innervated by bipolar tail neurons with central axons that extend to reach the visceral ganglion. Expression of Neurog and of the Atoh1/7-pro-ortholog (Atonal) is involved in the development of these cells. Vertebrates have sensory neurons located in peripheral sensory ganglia whose development depends on the expression of Neurog1 followed by Neurod1; these have peripheral axons that either may or may not contact secondary sensory cells, and central axons that project into the spinal cord and the brainstem. The brainstem receives afferent input from electroreceptor (ELL) and lateral line (LL) sensory neurons that connect peripherally to Atoh1+ secondary sensory (hair) cells in the body wall, and from auditory and vestibular sensory neurons that contact hair cells in the inner ear (gray), which is unique to vertebrates. Modified after (Tang et al., 2013; Stolfi et al., 2015; Holland and Daza, 2018; Manni et al., 2018; Holland, 2020; Zhao et al., 2020; Elliott et al., 2021; Fritzsch, 2021).

Lancelets have primary sensory cells (sensory cells with axons that project to the CNS) widely distributed in the skin (Poncelet and Shimeld, 2020; Holland and Holland, 2022). Among these, a unique group in the corpuscles de Quatrefages extend neurites into the dorsal entry points of the first and second nerves (Fritzsch, 1996; Wicht and Lacalli, 2005). In addition, there are two types of widely distributed solitary sensory cells in the skin, type I and type II [Figure 4; (Wicht and Lacalli, 2005; Schlosser, 2021a)], each with a central kinocilium surrounded by microvilli, which are branched in the type II sensory cells. The type I sensory cells are primary sensory cells. The type II sensory cells have been suggested to be secondary sensory cells (sensory cells that do not have a centrally projecting axon) with short basal processes (Wicht and Lacalli, 2005) (Figure 3). Although the type II sensory cells are well positioned for mechanoreception and on morphological grounds have the appearance of hair cells, it is not yet clear whether they function as hair cells. Extensive RNA sequencing characterization has shown that the origin of the lancelet epidermal sensory cells is inconsistent with homology to neural crest-derived sensory ganglion neurons of vertebrates (Schlosser, 2021; Chowdhury et al., 2022; Zine and Fritzsch, 2023); indeed, there are no overt ganglia associated with lancelet afferent nerves (Fritzsch and Northcutt, 1993). Further work is needed to assess whether the origin of the type II sensory cells is homologous to the vertebrate placodes that give rise to the vestibular, auditory or lateral line hair cells.

In tunicates, peripheral ciliated sensory neurons have been described at larval and adult stages in ascidians (such as *Ciona*), thaliaceans and appendicularians (such as *Oikopleura*). In *Ciona*, these include the larval caudal and trunk epidermal neurons (CENs and TENs) and the papilla neurons (PNs). CENs and TENs are ciliated and have axons extending to the nerve cord, and thus appear to be primary sensory cells. CEN and TEN axons contact each other and/or central bipolar relay neurons in the CNS [Figure 4; (Tang et al., 2013; Stolfi et al., 2015; Manni et al., 2018; Ryan et al., 2018; Piekarz and Stolfi, 2023)]. In ascidians the CENs and TENs are positioned peripherally along the length of the body in a way that is reminiscent of the vertebrate lateral line, and they originate from neurogenic regions of the ectoderm (the neurogenic midlines) that are reminiscent of the postotic placodes that give rise to lateral line neuromasts and sensory neurons in teleosts. However, since they appear to be primary sensory cells, the potential homology would be to the sensory neurons in the lateral line ganglia, not to the hair cells of the lateral line neuromasts.

Despite the connectional similarity between the central bipolar neurons in *Ciona* (that synapse with the CENs and TENs) and vertebrate sensory neurons in dorsal root, vestibular and cochlear ganglia, it is unclear whether these are homologous [Figure 4; (Schlosser, 2021a; Fritzsch, 2021)]. Moreover, a developmental origin in tunicates homologous to the otic placode that gives rise to the vertebrate inner ear and most of the neurons in its associated ganglia (Schlosser, 2021; Zine and Fritzsch, 2023) has not been demonstrated.

The PNs are ciliated peripheral neurons that extend axons towards the sensory vesicle. At early larval stages some PNs synapse onto RTENs peripheral to the sensory vesicle; it is unclear whether later growing PN axons do the same or extend into the sensory vesicle (reviewed in Anselmi et al., 2024, in press). PNs have a combined chemosensory and mechanosensory function, responding to chemical cues that are known to promote substrate attachment, and to mechanical stimulation which upon substrate contact triggers metamorphosis to the adult sessile stage (Hoyer et al., 2024). The PNs develop from the anterior neurogenic zone.

The appendicularian tunicate *Oikopleura* bears a bilateral pair of secondary sensory cells (the Langerhans receptors) each with a long, modified ciliary structure. These have been shown to have a mechanosensory function (Bone and Ryan, 1979; Holmberg, 1986), and are therefore potentially homologous to vertebrate hair cells. But their embryonic origin and molecular patterning have not been assessed.

Ascidian, thaliacean and appendicularian tunicates also have ciliated secondary sensory cells in the circumoral region in free-swimming forms (larval ascidians and thaliaceans and larval and adult appendicularians), and in the coronal organ in both free-swimming (appendicularian) and sessile and pelagic (ascidian and thaliacean) adult forms (Engelmann and Fritzsch, 2022) (reviewed in Anselmi et al., 2024, in press). These are potentially homologous to vertebrate hair cells. However, there is great diversity among tunicate species in the organization of cilia in these cells, which is different from that of vertebrate hair cells in the inner ear and lateral line. The secondary sensory cells of the tunicate coronal organ have a central kinocilium with surrounding stereocilia, whereas the cilia bundle of vertebrate hair cells has a distinct axis with the kinocilium at one extreme and stereocilia arrayed in decreasing lengths towards the other extreme (the kinocilium subsequently degenerates in mammalian cochlear hair cells, but the axis of graded stereocilia height remains). In both tunicates and vertebrates the cilia are bound to each other by link proteins, strongly indicative of a similar mechanosensory function. Indeed, in some tunicate species, the secondary sensory cells in the coronal organ have been demonstrated physiologically to have a mechanoreceptive function, but it is unclear whether all are mechanoreceptive or alternatively chemoreceptive (or both) (Caicci et al., 2013; Manni et al., 2018; Poncelet and Shimeld, 2020; Engelmann and Fritzsch, 2022; Van Le et al., 2023) (reviewed in Anselmi et al., 2024, in press). A cladistic analysis has suggested a monociliated cell as the ancestral form giving rise to the diverse forms exhibited in tunicate coronal organs (Rigon et al., 2013).

The coronal organ derives from the stomodeal portion of the anterior proto-placode in tunicates, a structure that has been suggested to be potentially homologous to one or more of the posterior vertebrate placodes (otic and/or lateral line) that give rise to hair cells. However, this potential homology remains to be proven, as there are some discrepancies in gene expression relative to vertebrate placodes during their development (reviewed in Anselmi et al., 2024, in press).

In vertebrates, the *Atoh1* gene plays a pivotal role in specifying the hair cell phenotype. A single *atonal/lin-32/Atoh* bHLH gene is found in Protostomia (fly*, D. melanogaster;* nematode*, C elegans*). This gene has been conserved in Deuterostomia, and duplicated in vertebrates to generate the genes *Atoh1* and *Atoh7*. In lancelets and tunicates, potential orthologs are the *NeuroD/Atonal-related* gene and the single *Atonal* gene, respectively. *Atonal* is expressed in the coronal organ of tunicates (Rigon et al., 2018). Of additional relevance with respect to sensory cell and neuron diversity are the genes *Neurod* (4 in vertebrates, 1 *NeuroD/Atonal-related* in lancelets, two potential orthologs in *Ciona*), *Neurog* (3 in vertebrates, 1 in lancelets, 1 in *Ciona*), and *Olig* (3 in vertebrates, 2 in lancelets, evidently absent in *Ciona*) (Simionato et al., 2007; Negrón-Piñeiro et al., 2020b). In vertebrates, *Neurog1/2* and *Neurod1* are associated with the emergence of distinct peripheral sensory cells (under the control of *Atoh1*) and of sensory neurons in sensory ganglia (direct action of *Neurog1/2 and Neurod1*) (Fritzsch, 2021).

Expression of *NeuroD/Atonal-related* in the lancelet nervous system has not been described (Simionato et al., 2007; Holland et al., 2008; Holland, 2020; Zhao et al., 2020; Chowdhury et al., 2022). In tunicates, *Neurog* and *Atonal* are expressed in the nerve cord and peripheral sensory cells (Tang et al., 2013; Stolfi et al., 2015; Cao et al., 2019; Ryan and Meinertzhagen, 2019). It is currently not known whether the specification of secondary mechanosensory cells (both putative and definitive) in tunicates depends on expression of *Atonal* in the way the specification of hair cells depends on *Atoh1* in vertebrates.

In summary, there are clear distinctions among putative mechanosensory cells and their sources of innervation in lancelets (epidermally derived primary sensory cells that project to the CNS), tunicates (epidermally derived primary sensory cells that project to the CNS and epidermally derived secondary sensory cells innervated by neurons in the CNS), and vertebrates (placode-derived secondary sensory cells that are innervated by placode- and neural crest-derived sensory neurons within sensory ganglia). *Atoh* gene ortholog expression appears to be a common feature in tunicate and vertebrate mechanosensory cells (*Atonal* in tunicates, *Atoh1* in vertebrates), but it is unclear if this is shared by lancelets. The recruitment (in some cases preceded by duplications) of additional genes governing differentiation of mechanoreceptors and associated sensory neurons is related to the more complex organization of mechanosensory systems in vertebrates, exemplified by the highly specialized vestibular and cochlear organs and their ganglia (Figure 4). A central element in this evolutionary elaboration has been the creation in vertebrates of dedicated placodal anlage (otic placode for auditory and vestibular, post-otic for lateral line) that give rise to peripheral mechanosensory structures bearing hair cells.

## Molecular specification of motoneurons and diversity of neuromuscular organization

In the vertebrate posterior neural tube, sonic hedgehog (SHH) acts as a graded morphogen emanating from the notochord and floor plate to promote the formation of ventral progenitor domains, one of which (pMN) gives rise to all motoneurons. WNT proteins and bone morphogenetic proteins (BMPs) emanating from the dorsal neural tube generate an opposing gradient that inhibits ventral progenitor formation and MN differentiation (Roure et al., 2023). The action of these opposing gradients, coupled with reciprocal repressive interactions between progenitor domain-defining transcription factors, leads to well defined and restricted progenitor populations, and is the basis for the localization of MNs in the ventral neural tube [later migrations can posit some MNs more dorsally; (Briscoe and Novitch, 2008)]. Except for the p3 ventral progenitor domain (which gives rise to V3 interneurons), the action of SHH is not a direct induction of ventral progenitor fates, but rather inhibition of the intracellular protein GLI3, which acts to repress ventral progenitor fates. It has been shown that MNs can still be generated in the absence of SHH and GLI3, indicating that MNs can be specified through another pathway that is normally repressed by GLI3. In this case, however, MNs and other (interneuronal) cell type precursors are intermingled instead of being organized into discrete anatomical populations. It has been proposed that the SHH signaling pathway has achieved a necessary status during vertebrate evolution to ensure that ventral progenitor domains are properly patterned in the face of increasing neuronal numbers.

Once specified, differentiation of MNs in vertebrates involves the expression of several genes, including the transcription factor genes *Nkx6.2*, *Olig*, *Isl1*, and *Mnx*, and culminating in the expression of choline acetyltransferase (ChAT) and the vesicular acetylcholine (ACh) transporter (vAChT), key components of the machinery for synthesizing and utilizing ACh as a neurotransmitter.

Lancelets and tunicates express hedgehog (*Hh*) genes homologous to vertebrate *Shh* (1 gene in lancelets, 2 in *Ciona*), as well as *Wnt* and *Bmp* genes dorsally (Shimeld, 1999; Lin et al., 2009; Hudson et al., 2011; Ryan et al., 2018; Benito-Gutiérrez et al., 2021). In both lancelets and vertebrates, HH/SHH is expressed ventrally (in both notochord and floor plate) together with *Patched* (PTC) and *Smoothened* (SMO), transmembrane proteins that transduce the HH/SHH signal, and with GLI and SUFU proteins that mediate SHH-elicited transcriptional events. However, in *Ciona* expression of *Hh1* and *Hh2* is spatially limited to the ventral nerve cord (absent in the notochord) and temporally limited there to early (pre-hatching) stages. PTC, SMO and GLI are also expressed, indicating that the main components of the HH-signaling pathway are present during the generation of MNs (Imai et al., 2009; Islam et al., 2010). However, MNs are still generated in the absence of *Hh* expression, suggesting that MN specification relies on a non-HH-mediated removal (spatially or temporally) of *Gli* expression (Hudson et al., 2011; Di Gregorio, 2020; Ren et al., 2020). This is similar to the situation created by double knockout of *Shh* and *Gli3* in vertebrates. Since only a handful of MNs are generated in tunicates, the role of *Shh/Hh* in creating a dorsoventrally restricted domain of MN progenitors may not be as important in tunicates as in vertebrates, and therefore may have been relaxed to the point that HH-signaling has been eliminated as a necessary factor for MN specification.

Lancelets have two types of motoneurons, dorsal (branchial) and ventral (somatic) (Fritzsch and Northcutt, 1993; Lacalli and Kelly, 2003; Wicht and Lacalli, 2005). There is a noticeable left/right asymmetry and an incomplete overlap of most rostral motoneurons (Ren et al., 2020). Lancelets have a single *Nkx6* gene, two *Olig* genes and a single *Islet* gene (Jackman et al., 2000; Ren et al., 2020), which probably have comparable functions to the mammalian orthologues (Delás and Briscoe, 2020). Both MN types express *Nkx6, OligA* and *Islet*, but the latter does not appear to be expressed in more posterior MNs (Jackman et al., 2000; Ren et al., 2020). As in vertebrates, MN specification in lancelets also involves *Hox* and *bHLH* genes, although the relevant interactions among these have not been well characterized in lancelets (Ferrier et al., 2001; Langeland et al., 2006; Leung and Shimeld, 2019; Dasen, 2022). In tunicates, which lack an *Olig* gene, the expression patterns and functional roles of *Nkx6*, *Islet*, and *Hox* orthologs in the context of MN differentiation have yet to be elucidated (Roure et al., 2023). Thus, it remains to be seen how similar this process is molecularly in the three chordate groups.

In lancelets, innervation of muscle is unusual, in that MNs do not project axons out of the spinal cord, but rather make contacts with muscle cells that appose the ventrolateral margin of the spinal cord (Flood, 1966; Wicht and Lacalli, 2005; Fritzsch et al., 2017). In tunicate larvae, MNs send axons into the periphery from the nerve cord. The ascidian tunicates *Ciona* and *Halocynthia* have at larval stages respectively 5 pairs of MNs and 3 pairs of MNs in the visceral ganglion, considered to be homologous to the posterior part of the vertebrate hindbrain (Dufour et al., 2006; Stolfi et al., 2015; Ryan and Meinertzhagen, 2019; Fritzsch and Martin, 2022). Since most of the tail musculature is located well caudal to these MNs, activation of the musculature requires that MN axons descend for multiple segments along the nerve cord prior to exiting into the periphery, and that more rostral muscle cells transmit depolarization to more caudal muscle cells via gap junctions (Okada et al., 2002). By contrast, in the appendicularian tunicate *Oikopleura*, there are 10 pairs of MNs corresponding to the 10 pairs of muscle cells, 3 in the caudal ganglion (roughly homologous to the visceral ganglion of ascidian tunicates) and 7 in the caudal nerve cord. Most of the MNs innervate a muscle cell at the same or next caudal segmental level. This near co-distribution of MNs and muscle cells along the rostrocaudal axis is more similar to the situation in vertebrates (Søviknes et al., 2007).

Extraocular muscles (EOMs) are in many ways unique compared to other skeletal muscles in vertebrates (Noden and Francis-West, 2006; Spencer and Porter, 2006; Fritzsch et al., 2017; Ziermann et al., 2018). They derive from epithelial mesodermal coeloms termed head cavities. Three pairs of head cavities form from the pharyngeal pouches, thereby exhibiting a metameric arrangement that has prompted the notion of three cranial somites (Bjerring, 1978; Gilland and Baker, 2005; Kuratani and Adachi, 2016). Extraocular muscles thus form relatively near the midbrain (near the source of cranial nerve III, the oculomotor nerve), the midbrain-hindbrain transition (near the source of cranial nerve IV, the trochlear nerve) and the hindbrain (the source of cranial nerve VI, the abducens nerve). Oculomotor and trochlear MNs are special somatic motoneurons (SSM, whose specification require expression of *Phox2a*), whereas abducens MNs are somatic motor neurons (SM, specification independent of *Phox2a*) (Epstein et al., 1999; Brunet and Pattyn, 2002; Litingtung et al., 2002; Fritzsch et al., 2017; Delás and Briscoe, 2020). Along with most (but not all) MNs in the hindbrain, differentiation of the SSM in the oculomotor and trochlear nuclei depends on the expression of *Phox2* transcription factors [reviewed in (Fritzsch et al., 2017)].

The development of extraocular muscles and their innervating MNs is a vertebrate innovation, arising through the need to mobilize the eye to create a more flexible and powerful visual system [reviewed in Fritzsch et al., 2024 in press]. Neither extraocular muscles nor MNs to innervate them are present in lancelets or tunicates. It is unclear whether lancelets express *Phox2* genes in the nervous system. Although *Phox2* is expressed in the ciliomotor neurons that control branchial ciliary flow in the sessile adult *Ciona* (which derive from the larval “neck” region), it is unclear which brain stem neurons these might correspond to in vertebrates. It is also unclear whether *Phox2* expression is involved in the differentiation of the larval MNs in the visceral ganglion, which also corresponds to part of the vertebrate brain stem (Gigante et al., 2023; preprint). Thus, SSM in general may be a vertebrate invention, beyond the specific lack of extraocular MNs in lancelets and tunicates (Fritzsch et al., 2017).

In summary, the different chordate taxa share many elements of a common molecular program for specifying generic MNs, but differences exist in the way specific genes and signaling pathways are utilized. In addition, a variety of neuromuscular organization patterns, including the direct apposition of muscle to the ventrolateral spinal cord obviating the need for peripheral MN axons in lancelets, and the rostrally restricted MN localization with activation of muscle by descending MN axons combined with sequential activation of more caudal muscle cells via gap junctions in ascidian tunicates, deviate substantially from the vertebrate pattern. On the other hand, the numerical equivalence and co-distribution along the anteroposterior axis of MNs, muscle cells and neuromuscular junctions in the appendicularian *Oikopleura* bears a close resemblance to the vertebrate organization, in which MNs are distributed along the entire anteroposterior axis of the brain stem and spinal cord in rough alignment with the skeletal musculature they innervate. The difference between ascidian and appendicularian tunicate patterns is almost certainly related to the rapid metamorphosis of ascidians into sessile adults contrasting with the continuous free-swimming lifestyle of the appendicularians. A major evolutionary advance in vertebrates has been the elaboration of MN subtypes generated by the incorporation of additional MN-specifying genes including the *Phox2* genes. This has clearly involved a co-evolution of more diverse skeletal muscle functions in vertebrates such as the deployment of extraocular muscles to generate eye movements.

## Summary and conclusion

Despite their differences in neural architecture, and the evolutionary distance separating them (about 600 million years), the three main chordate taxa should exhibit some degree of homology in the development and organization of major sensory and motor cell types. Current knowledge regarding the molecular underpinnings of chemo-, photo- and mechanosensitive cells and organs, and of motoneurons, indeed reveals common genetic elements in lancelets, tunicates, and vertebrates. These include the expression of a family of diverse OR genes in lancelet chemosensory cells and the vertebrate olfactory epithelium, the link between *Pax4/6* and photoreceptive cells in lancelets, tunicates and vertebrates, the utilization of *Atoh1/7* genes in specifying ciliated secondary sensory cells in tunicates and hair cells in vertebrates, and the pivotal role of the SHH-signaling pathway in generating MN progenitors in all three taxa. On the other side of the coin, significant differences in the presence or utilization (expression pattern) of specific genes in lancelets and tunicates are related to an unelaborated olfactory system (lack of *Foxg1* expression in CNS of tunicates), the primitive nature of their eyes (lack of expression of *Atoh1/7-*like genes in lancelets and tunicates), the less elaborate organization of their mechanosensory systems (lack of an otic placode in lancelets and tunicates) and less MN diversity (lack of *Phox2* recruitment into MN differentiation in lancelets and tunicates). Two major innovations that likely have been major drivers of the differences between the protochordate taxa and vertebrates are the extended repertoire of transcription factors (enabled by WGD) employed in specifying sensory and neuronal cell types, and the introduction of placodal anlage that elaborated the peripheral structures associated with olfaction, vision and mechanoreception.

Further study of the gene networks responsible for protochordate sensory and motor development should gradually fill in the many gaps that remain in the comparative assessment of homology and diversity of protochordates relative to vertebrates. In this regard, it is important to point out that our current knowledge is based on the investigation of only a handful of the 30 or so extant species of lancelets (which have similar body plans and lifestyles) and the approximately 3,000 extant species of tunicates (which have quite diverse body plans and lifestyles). The far greater number of vertebrate species (about 60,000) are arguably better represented by the relatively few model organisms that have been investigated extensively, but also here there are likely to be interesting examples of diversity not yet revealed, which will necessitate explanation at the molecular level.
